# AAV library screening identifies novel vector for efficient transduction of human aorta

**DOI:** 10.1038/s41434-024-00511-8

**Published:** 2024-12-18

**Authors:** Lena C. Schröder, Leonard Hüttermann, Anca Kliesow Remes, Jakob C. Voran, Susanne Hille, Wiebke Sommer, Georg Lutter, Gregor Warnecke, Derk Frank, Dennis Schade, Oliver J. Müller

**Affiliations:** 1 Department of Internal Medicine V, University of Kiel, Kiel, Germany; 2 German Centre for Cardiovascular Research, Partner Site Hamburg/Kiel/Lübeck, Kiel, Germany; 3 Department of Internal Medicine III, University of Kiel, Kiel, Germany; 4 Department of Cardiac and Vascular Surgery, University of Kiel, Kiel, Germany; 5 Department of Pharmaceutical & Medicinal Chemistry, Institute of Pharmacy, University of Kiel, Kiel, Germany

**Keywords:** Cardiovascular diseases, Genetic vectors

## Abstract

Targeted gene delivery to vascular smooth muscle cells (VSMCs) could prevent or improve a variety of diseases affecting the vasculature and particularly the aorta. Thus, we aimed to develop a delivery vector that efficiently targets VSMCs. We selected engineered adeno-associated virus (AAV) capsids from a random AAV capsid library and tested the top enriched motifs in parallel screening through individual barcoding. This approach allowed us to distinguish capsids that only transduce cells based on genomic DNA (gDNA) from those also mediating transgene expression based on transcribed cDNA reads. After three rounds of selection on primary murine VSMCs (mVSMCs), we identified a novel targeting motif (RFTEKPA) that significantly improved transduction and gene expression efficiency over AAV9-wild type (WT) and increased expression in mVSMCs by 70% compared to the previously identified SLRSPPS peptide. Further analysis showed that the novel motif also improved expression in human aortic smooth muscle cells (HAoSMCs) and human aortic tissue ex vivo up to threefold compared to SLRSPPS and approximately 70-fold to AAV9-WT. This high cross-species transduction efficiency makes the novel capsid motif a potential candidate for future clinical application in vascular diseases.

## Introduction

Vascular smooth muscle cells (VSMCs) play a critical role in maintaining the structural and functional integrity of blood vessels [1, 2]. They play a critical role in regulating vascular tone, blood pressure, and overall cardiovascular homeostasis. Dysregulation of VSMC function is implicated in various vascular disorders, including atherosclerosis, hypertension, and aortic aneurysms [3, 4]. Considering their crucial role in developing vascular lesions and remodeling, VSMCs are a promising target for gene therapy approaches.

Adeno-associated virus (AAV)-based vectors have been widely used in clinical studies and are increasingly also considered for preclinical studies in the vascular system predominantly using skeletal muscle gene transfer in peripheral artery disease [5, 6]. While the AAV9-based vector Zolgensma has already been approved for gene therapy into skeletal muscle in pediatric muscular atrophy patients [7], transduction of aortic VSMCs is low in vivo and in vitro with all naturally occurring AAV serotypes [8–10]. We have previously enhanced the efficiency of AAV9 vectors in transducing endothelial cells (EC) using a random AAV capsid library [11]. This library is based on the insertion of an oligonucleotide library into the AAV cap gene, leading to the display of randomized heptapeptides on the viral capsid surface, thus altering viral tropism. Using highly diverse random libraries potentially improves the transduction efficiency and specificity toward the targeted cell type. Over the past decade, ECs have been in focus for gene transfer into vascular tissue [10–14]. Considering that VSMCs play a predominant role in various vascular pathologies, developing a VSMC-directed AAV could improve gene transfer to the walls of large vessels, including the aorta. For this, we chose the AAV9- wild type (WT) as the baseline vector for our selections, as we had already generated a highly efficient vector from this serotype (SLRSPPS) [11], and it is suitable for translation to clinical application.

In this study, we present novel AAV vectors selected from a randomized AAV9 library to enhance transduction efficiency in VSMCs in vitro and human aortic samples ex vivo. Therefore, our study sets the stage for further exploration of these AAV variants in large animal models using local delivery methods, aiming to advance personalized therapeutic strategies in vascular diseases.

## Materials & methods

### AAV production

HEK293T cells were transfected with 3 different plasmids using polyethylenimine (PEI; Polysciences, Warringtion, Pennsylvania), and AAVs were harvested after 3 days. Purification and titration were performed according to standard procedures [15]. For the production of barcoded AAV genomes, the pscAAV-CMV-eYFP-BC-bGHpolyA was used as genome plasmid and was kindly provided by the Grimm lab (Heidelberg, Germany [16]). The desired capsid motif is determined by the p5E18vd2/9-SfiI1759 helper carrying rep/cap genes and lacking inverted terminal repeats (ITRs) [11]. In this plasmid, the cap9 gene contains *Sfi* restriction sites for cloning the heptamer insertion at the A589 position. Production of barcoded WT AAVs of the serotypes 4, 5, 6, and 9 as well as benchmark controls was performed with the respective rep/cap helper plasmid [17]. The pDGΔVP is lacking the cap gene and was used for its adenovirus helper functions in all productions [18]. Ten 15 cm dishes with 5.4 × 10^6^ cells/dish were used for separate production of each capsid variant. To generate a random AAV library, the library plasmid backbone pKV-AAV9Lib/BB was used as genome plasmid [11]. One library was produced in 40 × 15 cm dishes of HEK293T cells.

### Cloning of random plasmid library

The pKV-AAV9Lib/BB carries the *cap9* gene within ITRs and is packed accordingly in the genome for expression and capsid building. Insertion of random (NNK)_7_ sequences was introduced in the *SfiI* restriction site. Ligation of the double stranded (NNK)_7_ oligonucleotide and *SfiI* digested backbone was performed at 1:30 molar ratio and transformed into electro-competent 10-beta *E. coli* (NEB, Frankfurt am Main, Germany). After overnight incubation at 37 °C and 225 rpm in MaxQ 8000 incubator shaker (Thermo Fisher Scientific, Waltham, Massachusetts), the plasmid library was purified with the Plasmid Plus Maxi Kit (QIAGEN, Hilden, Germany) and used for AAV library production.

### Subcloning of enriched variants

Isolated gDNA and cDNA of mVSMCs after each selection round contained internalized vector genomes that were used for production of the selected AAV library for the next infection cycle. For this, the insertion region of the *cap9* was PCR-amplified and inserted into the p5E18vd2/9-SfiI1759 plasmid backbone generating a plasmid library which was used for production of a the selected AAV library.

### Cell culture

Murine vascular smooth muscle cells (mVSMCs) were isolated from adult murine aorta of both sexes (C57BL/6). Permission for organ removal was granted by the animal ethics board of the Ministry of Energy, Agriculture, the Environment, Nature and Digitalization Schleswig-Holstein (MELUND, V312-7224.121-4). Randomization and blinding were not performed nor required. Isolated aortae were flushed with PBS and cut into 2 mm^2^ pieces. Next, mVSMCs were isolated via 6 h incubation with 0.1% collagenase type II (Worthington Biochemical Corporation, Lakewood, New Jersey) at 37 °C. mVSMCs were routinely stained with smooth muscle actin (SMA) marker in order to prove the purity of the primary cells. Murine cardiac fibroblasts were isolated from murine adult heart. Isolated heart was cut into 2 mm^2^ pieces and incubated with collagenase type II and DNase I (Sigma-Aldrich, St. Louis, Missouri) for 45 min at 37 °C under continuous shaking. NRVCMs were isolated from neonatal rat hearts as previously published [19]. All cell types were maintained in Dulbecco’s modified Eagle’s medium (Sigma-Aldrich, Hamburg, Germany) supplemented with 10% heat-inactivated fetal bovine serum (FBS; PAA, Coelbe, Germany), 2 mM L-glutamine (Gibco, Karlsruhe, Germany), and 1 x Pen/Step (Life Technologies, Carlsbad, California).

HAoSMC and HUVECs were provided cryo-preserved by Promocell and maintained in the recommended medium supplied by the company (Promocell, Heidelberg, Germany).

For passaging and seeding of all cell types 0.05% Trypsin-EDTA (Gibco, Karlsruhe, Germany) was used. Viral transduction was performed overnight in serum-free medium. Cells were further cultured for 3 days in 10% FBS-supplemented medium.

### Animal study

Animal experiments were carried out under the guidelines from directive 2010/63/EU of the European Parliament on the protection of animals used for scientific purposes with the approval of the regional council (MELUND, permission no. V242-56596/2022). Animals were housed under standard conditions with a 12h-light- / 12h-night-cycle. Water and food were offered ad libitum. Barcoded AAV vector libraries were injected systemically via tail vein injection into eight-weeks-old male C57BL/6 N mice (Charles River). Each mouse was injected with 5 × 10^11^ vg (viral genomes) in a total volume of 100 µl. Mice were sacrificed two weeks after injection by cervical dislocation and harvested organs were snap-frozen in liquid nitrogen.

### Transduction of human aortic tissue

Permission for using aortic tissues from patients undergoing surgery at the University Hospital Schleswig-Holstein, Kiel, Germany was granted from the Ethical Board of the Medical Faculty of the University of Kiel (permission no. P2N 2024-011). Informed consent was obtained from all patients. Tissue was collected, cut into 3–5 mm^2^ pieces, and maintained in Ham’s F12 Medium (Corning, Glendale, Arizona) supplemented with 10% FBS and 1× Pen/Strep as previously described [20]. AAV transduction with 10^10^ vg for RNA quantification was performed overnight in serum-free medium followed by 3 days of incubation in FBS-supplemented medium. For protein determination tissues were incubated with 5 × 10^10^ vg for 4 days. Injection of 10^10^ vg into the tissue were performed with insulin injections in serum-free medium and medium was changed after overnight expression to FBS-supplemented medium for further 3 days.

### Total DNA and RNA isolation and qRT-PCR

DNA and RNA were simultaneously extracted from cells or tissues with AllPrep DNA/RNA Mini Kit (QIAGEN, Hilden, Germany) according to manufacturer’s instructions. Before isolations, approx. 5 mg human aortic tissue per sample was snap-frozen and pulverized in liquid nitrogen using a pestle and mortar. Subsequently 1 µg RNA was transcribed to cDNA with LunaScript® Reverse Transcriptase SuperMix Kit (NEB, Frankfurt am Main, Germany) according to manufacturer’s instructions. For eYFP quantification in human aortic tissues, 12.5 µg cDNA was used for qRT-PCR with SensiFAST SYBR No-ROX Kit (Bioline, Luckenwalde, Germany) in a CFX96 real-time PCR detection system (Bio-Rad, Hercules, California). The following primers were used for eYFP quantification: forward primer: 5ʹ-GCATCAAGGTGAACTTCAAGATCC-3ʹ and reverse primer: 5ʹ-ATGTGATCGCGCTTCTCGTTG-3ʹ. EYPF values were normalized to human ribosomal protein, large, P0 (hRPLR0). The eYFP expression from a vector was indicated as x-fold over untransduced cells or tissues.

### NGS of amplified insertion region and barcodes

Region of interest of cDNA and gDNA samples was amplified by PCR reactions comprising Q5 High-Fidelity DNA Polymerase (NEB, Frankfurt am Main, Germany) to generate a 215 bp product of barcode regions and a 245 bp product for the insertion region in the *cap9* gene. The PCR reaction mix was purified with NucleoSpin Mini Kit (Macherey-Nagel, Düren, Germany) and amplicons were subsequently sent for deep sequencing via amplicon sequencing service provided by Genewiz (Leipzig, Germany).

### NGS data processing

For data processing, the python 2.7 script published by the Grimm lab [16] was applied to fastq files to count the 15 nt barcode sequences. The output files were used to normalize read counts and calculate the proportion of all variants in one tissue and the proportion considering the variation in the input library displaying the efficiency of individual variants on cells. For insert region amplicons, the configuration file of the script was adapted according to the flanking regions. The output provided the read counts of non-determent nucleotide sequences that were normalized on flow cell variation and ranked based on their frequency.

### Flow cytometry

For analysis of transduction efficiency, cells were harvested and washed with PBS. Cells were monitored in fluorescence-activated cell sorting in a Flow Cytometry Analyser (Institute for Immunology, UKSH Kiel) and the percentage of eYFP positive cells (transduced cells) out of 10,000 events in technical replicates was evaluated with BD FACSDiva software (BD Biosciences).

### Western blot analysis

Western blot analysis was performed according to standard protocols using 50 µg protein and antibody against GFP (1:1,000, 2956S, Cell Signaling, Danvers, Massachusetts) and corresponding secondary antibody anti-rabbit IgG-HRP (1:10,000, Santa Cruz Biotechnology, Dallas, Texas). Tubulin was used as housekeeping protein and detected with primary antibody (1:8000, MAB-9344-100, R&D Systems, Minneapolis, Minnesota) and secondary mouse IgG-HPR (1:10,000, Santa Cruz Biotechnology, Dallas, Texas). Chemiluminescence was detected with the Chemidoc™ MP Imaging System (Bio-Rad, Hercules, California). Densitometric analysis was performed using ImageJ software (version 1.54).

### Immunohistochemistry

Human aortic tissue pieces were fixed in 4% paraformaldehyde for 4 h at 4 °C prior to embedding and freezing in TissueTek®. 7 µm sections were blocked with 2.5% BSA solution containing 0.01% Triton-X-100 for 1.5 h. 647-conjugated anti GFP antibody (Invitrogen, Eugene, Oregon, catalog no. A31852) was diluted 1:500 in blocking buffer and used to incubate sections overnight at 4°C in a humidified atmosphere. Images were taken by a fluorescence microscope (Keyence BZ-X810, Neu-Isenburg, Germany).

### Statistics

Sample sizes were chosen based on previous experience [16]. Data are presented as means ± SD and analysed for statistical significance with ordinary one-way ANOVA with Tukey’s multiple comparisons for three or more groups. Calculation was performed in GraphPad Prism version 9.5.0 and *p* values are defined as <0.05 = *, <0.01 = **, <0.001 = ***, and <0.0001 = ****.

## Results

**Fig. 1:**
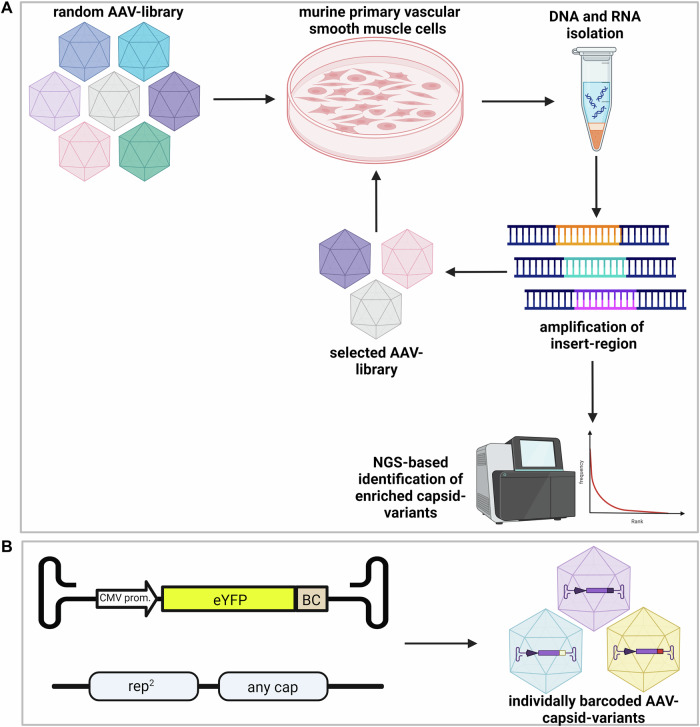
Overview of the procedure for AAV-library selection and validation of barcoded variants. **A** Selection process of random AAV-libraries. Primary isolated mVSMCs were infected with a random high-diversity AAV-library. DNA and RNA were isolated and the randomized region was amplified for NGS-based library analysis and variant detection and to clone back into the plasmid backbone for generation of AAV libraries for subsequent selection. **B** Individual barcoding of enriched capsid-variants allows for parallel testing of transduction and gene expression efficiency. AAV-genomes contain a CMV promoter driving an eYFP reporter gene followed by an individual barcode.

We generated a highly diverse AAV9 library according to a previously engineered plasmid construct, which allows the insertion of a random heptamer at amino acid A589 of the AAV9 cap gene [11]. The insertion site is displayed on the capsid surface and is likely to be involved in receptor binding and thereby might determine viral tropism. For the random AAV9 library, a complexity of 1.7 × 10^7^ different heptamer insertions at plasmid level was calculated based on bacterial colonies after transformation. Deep sequencing of the library revealed no unspecific enrichment of certain insertion sequences (Fig. S1). mVSMCs were infected with a multiplicity of infection (MOI) of 5 × 10^5^ vg per cell and DNA and RNA was isolated from cells. The capsid insertion region was amplified and used for generation of a selected library for reinfection (Fig. 1). In total, three selection rounds were performed with genomic DNA (gDNA). In addition, selection on the RNA level (as cDNA) was also included in the last round.

### Selection of the random AAV9 peptide library in mVSMCs resulted in enrichment of novel capsid-motifs

**Fig. 2:**
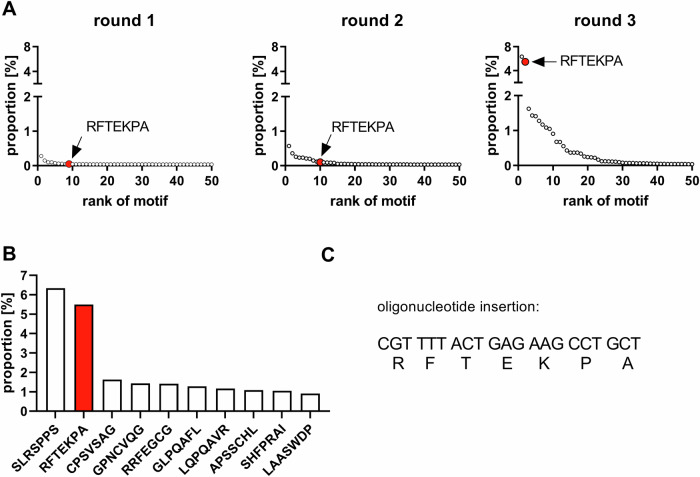
NGS-controlled in vitro selection of a random AAV9 capsid library on mVSCMs. **A** Proportion of the top 50 enriched motifs over three selection rounds. **B** Top 10 enriched peptide motifs after third selection round. **C** Nucleotide sequence encoding for the RFTEKPA peptide motif. Red dots and bar plots indicate RFTEKPA.

**Fig. 3:**
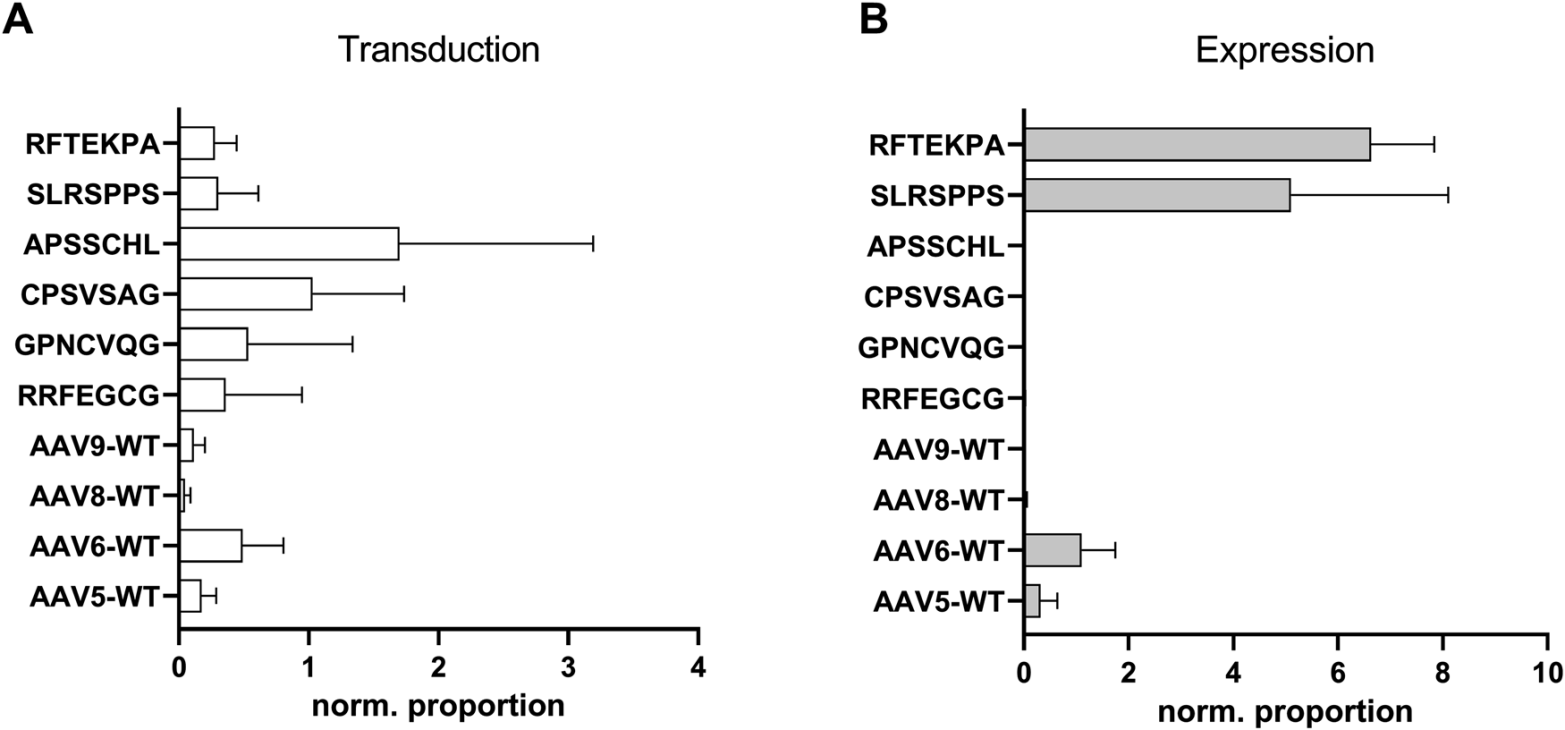
Parallel testing of barcoded AAV9 capsid variants on the efficiency of transduction (DNA) and expression (RNA) level on mVSMCs. The frequency of individual barcodes was determined using NGS, normalized for differences in abundance in the input library, and is presented as normalized proportion for transduction (**A**) and expression efficiency (**B**) for each variant. Expression mediated by RFTEKPA is massively increased over all tested WT AAVs (*p* ≤ 0.001) and higher or similar compared to previously identified SLRSPPS (*p* = 0.9) (*n* = 3, one-way ANOVA with Tukey’s multiple comparisons test).

The selection process revealed, amongst others, the RFTEKPA capsid motif, which massively increases transgene expression in mVSMCs. Sequencing of the insertion region after each selection round revealed an accumulation of distinct heptamers and allowed the enrichment of the RFTEKPA peptide to be observed (Fig. 2A). After the first selection round, RFTEKPA had a relative abundance of 0.05% in the AAV library and increased to 0.1% after the second selection round among 21,469 detected motifs. After the third selection round, the most prominent peptide was RFTEKPA, which comprised 5.5% of the library and was only exceeded by the previously identified SLRSPPS peptide (Fig. 2B) [11]. The potential of the top five enriched peptides was assessed by producing single viral vectors using the respective enhanced *cap9*-variant and a distinctive genome composed of a cytomegalovirus (CMV) promoter controlled enhanced yellow fluorescent protein (eYFP) reporter gene tagged in its 3’reagion by a unique barcode sequence assigned to the variant (Fig. 1). The barcode allows for parallel testing of the capsid variants compared to different WT AAV serotypes in a pooled barcoded library. After infecting mVSMCs with a MOI of 5 × 10^5^, we assessed transduction efficiency by measuring the vg count on gDNA level. RNA was reverse-transcribed to measure the expression potential of vectors (Fig. 3). Our results demonstrate that RFTEKPA revealed the highest expression comparable with SLRSPPS. Interestingly, all other vector variants tested in this screening transduced mVSMCs more effectively than RFTEKPA and SLRSPPS, but did not mediate transgene expression (Fig. 3). Transduction of the respective WT serotypes AAV9, AAV8, AAV6, and AAV5 were used as controls. As shown in Fig. 3A, AAV6 transduction efficiency was markedly increased as compared to the other WT AAVs. AAV6 also mediated highest transgene expression of all WT AAVs, followed by AAV5. In contrast, no expression was detected with AAV8- and AAV9-WT vectors (Fig. 3B). Moreover, we could demonstrate that the insertion of all novel capsid motifs improved the transduction of mVSMCs compared to AAV9. Furthermore, our data show that insertion of the RFTEKPA peptide massively increased transgene expression as compared to AAV9-WT (Fig. 3B).

### Individual in vitro studies of the RFTEKPA peptide confirms improved transduction efficiency and transgene expression in mVSMCs and other cell types of the cardiovascular system

**Fig. 4:**
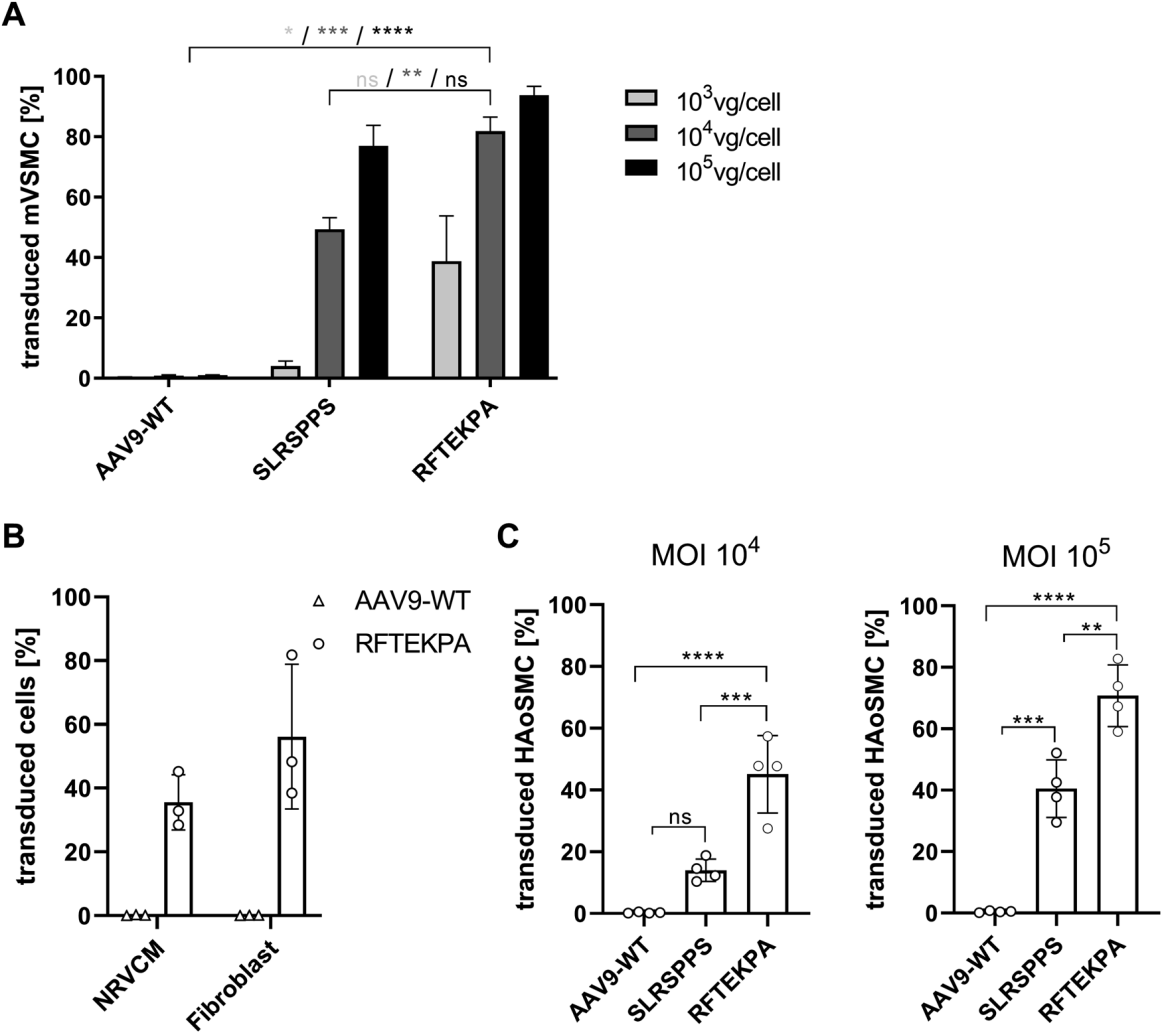
Characterization of the novel RFTEKPA variant in vitro. Cells were transduced with different AAV variants harboring an eYFP reporter gene. Expression of the eYFP reporter was measured using flow cytometry and depicted as transduceed cells [%]. **A** Gene expression efficiency of RFTEKPA was compared to AAV9-WT and SLRSPPS on mVSMC at different MOIs (*n* = 3). **B** Gene expression efficiency of RFTEKPA compared to AAV9-WT on neonatal rat ventricular cardiomyocytes (NRVCM) and murine fibroblasts (*n* = 3). **C** Gene expression efficiency of RFTEKPA was compared to AAV9-WT and SLRSPPS on human aortic smooth muscle cells (HAoSMC, *n* = 4, **p < 0.01, ***p < 0.001, ****p < 0.0001, one-way ANOVA with Tukey’s multiple comparisons test).

The result from the initial screening of the barcoded library was validated via individual testing of the vectors in flow cytometry, where transduced cells are identified via expression of the eYFP reporter. We compared gene expression efficiency of the RFTEKPA vector with SLRSPPS and AAV9-WT at MOIs of 10^3^, 10^4^, and 10^5^ (Fig. 4A). While AAV9-WT did not transduce mVSMCs at any MOI, flow cytometry analysis confirmed a massively increased transgene expression efficiency of the RFTEKPA peptide, e. g. 39% transduced cells at MOI 10^3^. This data confirmed the results from NGS-based screening of barcoded variants (Fig. 3B). Likewise, the remaining enriched peptides did not result in transgenic eYFP expression (Fig. S3). At MOI 10^4^ and 10^5^, transduction efficiency mediated by RFTEKPA was 82% and 94% and significantly increased over AAV9-WT at all MOIs. RFTEKPA transduction was also significantly increased over SLRSPPS at MOI 10^4^, which transduced 49% of the cells.

To rule out species-depended effects, RFTEKPA was also tested for gene expression into human aortic smooth muscle cells (HAoSMCs). As depicted in Fig. 4C, gene expression efficiency of RFTEKPA was significantly increased over AAV9-WT (by ~40-fold) and SLRSPPS (by ~3-fold) at MOI 10^4^ in HAoSMCs. SLRSPPS mediated only 14% eYFP positive cells that was not significantly more efficient than AAV9-WT (*p* = 0.069). Additionally, increasing the MOI to 10^5^ resulted in high gene transfer  by RFTEKPA to 71%, also outperforming AAV9-WT (0.5%) and SLRSPPS, whose transduction efficiency increased to 40%.

Considering its outstanding performance in SMCs, we investigated whether RFTEKPA allows transduction of further cardiovascular cell types. Neonatal rat ventricular cardiomyocytes and primary murine fibroblasts, cell types that comprise the major cellular part of the heart, were efficiently transduced (36% and 56%) by RFTEKPA at MOI 10^4^, while the respective WT AAV did not show transduction at all (Fig. 4B). To determine whether RFTEKPA is suitable for gene transfer into  vascular tissues in vivo using a systemic route of administration, a barcoded library comprising the enriched mutated capsids and a set of AAV wild-type vectors (AAV4, 5, 6, 8, and 9) and benchmark controls (AAV2-DJ, AAV2-ESGH, AAV9-RGDLGLS, and AAV9-SLRSPPS) was intravenously injected into mice with 5 × 10^11^ vg. Deep sequencing of aorta and other organs after 3 weeks of injection did not reveal an improved gene expression over AAV9-WT by any of the mutant vectors (Fig. S2).

Taken together, insertion of the RFTEKPA motif extends the ability of the AAV9 serotype to transduce murine and human SMCs in vitro and shows impressive transgene expression. Increased transduction efficiency of other cardiovascular cell types suggests RFTEKPA as a potent vector for in vitro transduction.

### Human aortic tissue is transduced by RFTEKPA ex vivo

**Fig. 5:**
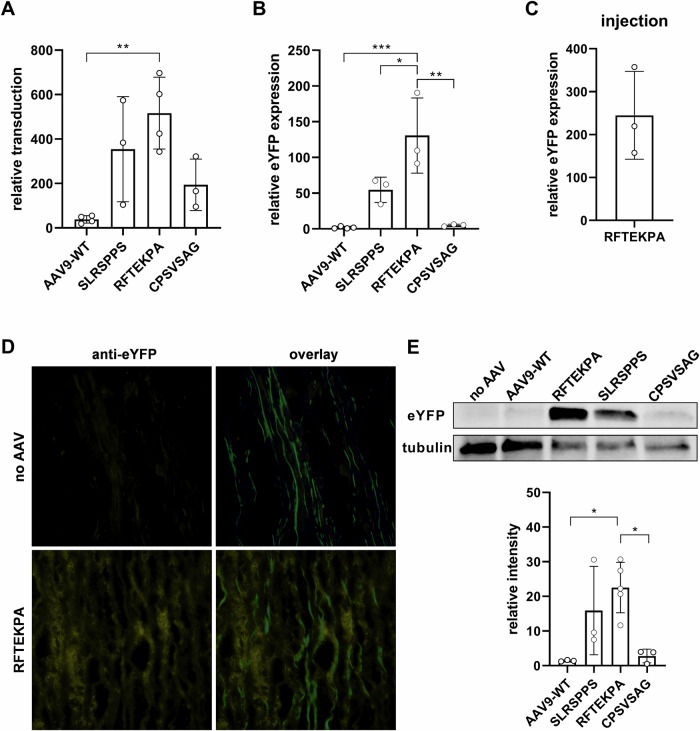
RFTEKPA transduces human aortic tissue ex vivo. **A** Viral transduction and (**B**) transgenic expression were analysed based on eYFP gDNA and RNA transcripts in qPCR. **C** Direct injection of 10^10^ vg RFTEKPA into aortic tissue (*n* = 3–4, *p < 0.05, **p < 0.01 ***p < 0.001, ****p < 0.0001, one-way ANOVA with Tukey’s multiple comparisons test). **D** Representative images of eYFP localisation of untransduced tissue vs tissue pieces incubated with RFTEKPA. eYFP was co-stained with 647-conjugated anti-GFP antibody (yellow) to distinguish between autofluorescence from elastin (green). **E** eYFP protein expression in relation to tubulin measured in Western blot (*n* = 3).

Efficient gene transfer into human SMCs indicates that RFTEKPA might also allows transduction of human vascular tissue upon local delivery. To test this, biopsies from human aorta were infected with AAVs and maintained 3–4 days ex vivo. qPCR analysis revealed significantly increased transduction from tissues infected with SLRSPPS and RFTEKPA over AAV9-WT, calculated from eYFP sequences detected on the gDNA level (Fig. 5A). Transduction from RFTEKPA was 38.5-fold increased over AAV9-WT. Quantification of eYFP transcripts via qPCR revealed a 75-fold increase in eYFP expression with RFTEKPA compared to AAV9-WT (Fig. 5B). Additionally, RFTEKPA expression was 2.4-fold higher than SLRSPPS. Direct injection of 10^10^ vg RFTEKPA into the aortic tissue revealed a high eYFP expression over untransduced tissue (Fig. 5C).

CPSVSAG is another peptide motif that accumulated over the selection process and was among the top three motifs after round three. Interestingly, insertion of this peptide into the AAV9 capsid did result in transduction, but not in transgene expression in mVSMCs (Fig. 3). Similarly, CPSVSAG transduction efficiency was increased in aortic tissue compared to AAV9-WT, but this capsid variant did not result in significant transgene expression (Fig. 5A, 5B). Moreover, we visualized reporter expression in the vascular wall of human vessels by incubating aortic sections with the RFTEKPA variant with an antibody against eYFP to distinguish between autofluorescence from elastin fibers in the green channel. As shown in Fig. 5D, we could observe a strong signal indicating efficient transduction of the complete vascular wall. Finally, Western blot analysis confirmed significant transgene expression from RFTEKPA and SLRSPPS but not from AAV9-WT and CPSVSAG (Fig. 5E).

## Discussion

Previously, an AAV9 library was successfully used to select a peptide on ECs that greatly increased transgene expression not only in endothelial but also in human coronary artery smooth muscle cells [11]. This study aimed to improve AAV9 transduction efficiency and transgene expression in particular in VSMCs by selecting a random AAV9 capsid library on VSMC. Selection of this library resulted in the identification of a peptide motif (RFTEKPA) that significantly enhanced transduction and expression. This was confirmed through NGS-based screening of barcoded variants and in in vitro characterization of individual capsid motifs.

We observed an accumulation of RFTEKPA in the AAV library over three selection rounds. The highest enrichment was observed after the third round, when RNA reads were included to produce the peptide library. Performing two rounds of selection applying only gDNA may have emphasized transduction and not necessarily gene expression as indicated by the low expression efficiency mediated by the third most frequent variant CPSVSAG. This was underpinned by the analysis of insert fragments from RNA, which indicated enrichment of the transcriptionally active SLRSPPS and RFTEKPA, but most of the further top candidates used in the screening could not be found from RNA reads (Table S1). It is tempting to speculate whether including RNA over the whole selection process would have yielded more vectors that mediate not only high transduction, but also efficient transgene expression. On the other hand, several previous studies revealed AAV capsid variants that lead to high transgene expression selected only on the gDNA [11, 21, 22]. The majority of NGS reads were related to SLRSPPS, confirming the previous enrichment in selection on endothelial cells. RFTEKPA was only the second most enriched peptide but resulted in the highest gene expression in VSMC, underlining the impact of careful characterization of individual variants.

Although murine cells were used for selection, the tropism of RFTEKPA for human cells was dramatically enhanced, which suggests that receptor binding and intracellular trafficking for AAVs in SMCs might be conserved in both species, at least for this variant. This is supported by the fact that the CPSVSAG peptide did not mediate transgene expression in mVSMCs and in human aortic tissue. The transduction efficiency on further non-smooth muscle cell types makes RFTEKPA a powerful tool in research, as it can be used for robust transgene expression in several cardiovascular cell types.

In the past, there was little focus on improvement of gene transfer into aortic SMC with capsid-modified vectors. Targeting ligands were identified on human saphenous vein SMCs using phage display in early studies [23]. The insertion of the EYHHYNK peptide into the AAV2 capsid increased transduction of human arterial SMCs [23]. Recent work identified the novel AAV-PR, which was originally selected from a random AAV9 library on murine brains [21]. In addition to brain vascular SMC, it transduces murine aortic VSMC after systemic injection. However, efficient transduction was only mediated with Cre expression in Ai9 mice and could not be verified in C57BL/6 and BALB/c mice with a GFP reporter.

Our study highlights, that selections from randomized AAV9 libraries generated from heptamer insertions into the capsid can enhance transduction of and gene expression in VSMC. However, systemic injection of the RFTEKPA vector in mice did not lead to transduction of aortic tissue which might be caused by first-pass liver transduction. Nevertheless, it was highly efficient in vitro, suggesting that the improved transduction properties could also depend on a local mechanism and a local delivery might be superior to systemic application. Successful ex vivo gene transfer in human aortic tissue indicates RFTEKPA as a potential vector for clinical applications to target vascular diseases. We have previously shown that SLRSPPS can be delivered to the murine aorta by implantation of vector-loaded alginate hydrogels [24]. In this study, RFTEKPA outperforms SLRSPPS in VSMCs and seems to transduce human aortic tissue more efficiently. In a future approach, it could be investigated whether gene transfer with RFTEKPA vectors is also enhanced over SLRSPPS vectors locally delivered with hydrogels. Moreover, we could show that reporter gene expression was extended across the whole aortic wall although the tissue was only briefly incubated in AAV-containing tissue culture medium confirming the ability of RFTEKPA vectors to mediate not only successful transduction but also efficient gene expression in human vascular tissue with the prospect for further clinical translation. A potential clinical application could be ex vivo transduction of vein grafts using RFTEKPA vectors for gene transfer approaches to prevent vein graft failure.

Such a local delivery strategy for aortic and vascular gene transfer might be advantageous in many ways. Is has emerged, that a major limitation of AAVs in gene therapy is the high vector doses required for intravenous injections [25]. Furthermore, systemic delivery resulted in major safety risks in clinical trials, due to AAV immunogenicity toxicities [26]. Finally, costly large-scale productions required for systemic delivery imply a burden for the health system. Thus, increased expression in HAoSMCs using RFTEKPA indicates the option to use low vector doses for treatment further reducing the risk for immunity and immune responses induced by AAV vectors [6, 27].

One limitation of RFTEKPA could be the lacking specificity for SMC. Over the recent years the use of tissue specific cis-regulatory elements was established for AAVs even within clinical trials [26]. In contrast to constitutively active promoters, specific regulatory sequences could reduce the risk for off-target effects, immune responses, and avoid excessive transgene overexpression resulting in cellular stress [28, 29]. In our study, a universal CMV expression-cassette was used, allowing unspecific and ubiquitous transgene expression that could be restricted to SMC by a tissue-specific promoter. In adult mammals, the smooth muscle α-actin (SM22α) gene is exclusively expressed in SMC-containing tissues and acts as a marker for differentiated SMCs [30–32]. In vitro studies confirmed cellular specificity of the SM22α promoter as transgenic mice or adenoviral transduction revealed gene expression restricted to SMC under control of this promoter [32, 33]. Additionally, enhancer elements could boost viral expression in SMC-rich tissues [34].

Taken together, we have identified a novel AAV9 capsid variant (RFTEKPA) that enables an efficient gene expression in VSMCs in vitro and in cultivated human vascular tissue ex vivo upon local application. To the best of our knowledge, this is the first AAV capsid variant systematically selected on VSMCs that could serve as a promising tool for future large animal studies with local vascular gene transfer.

## Supplementary information

Supplementary Information.

## Data Availability

The data that support the findings of this study are available from the corresponding author, [O.J.M.], upon reasonable request.
