# Supplementary material for: AAV library screening identifies novel vector for efficient transduction of human aorta
Source: Gene Ther. 2024 Dec 18;32(2):154–62. doi: 10.1038/s41434-024-00511-8 (PMC11946879; doi:10.1038/s41434-024-00511-8)
Supplement: Supplementary file 1 — Supplementary Information. [file 41434_2024_511_MOESM1_ESM.pdf]

Supplementary Information

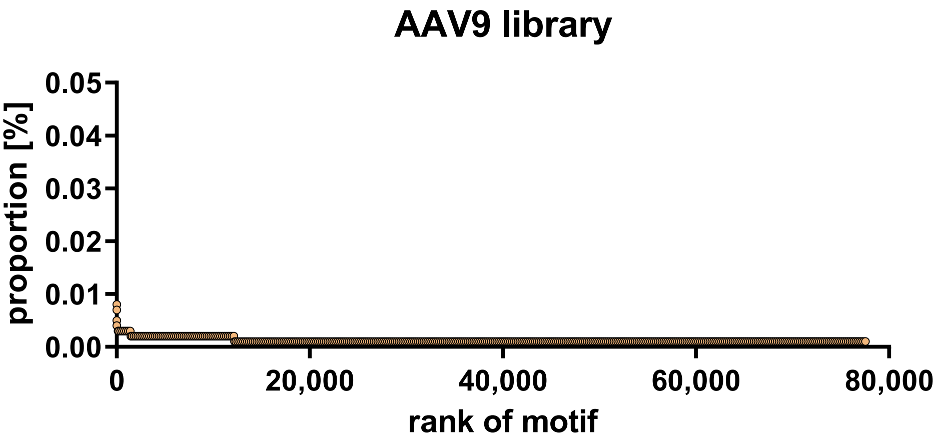

Figure S1. Quality control of initial AAV9 library. Deep sequencing of the insert region of the initial AAV9 library did not reveal unspecific enrichment before the first selection round. The sequencing depth of the amplicon sequencing was insufficient to depict the actual diversity of library.

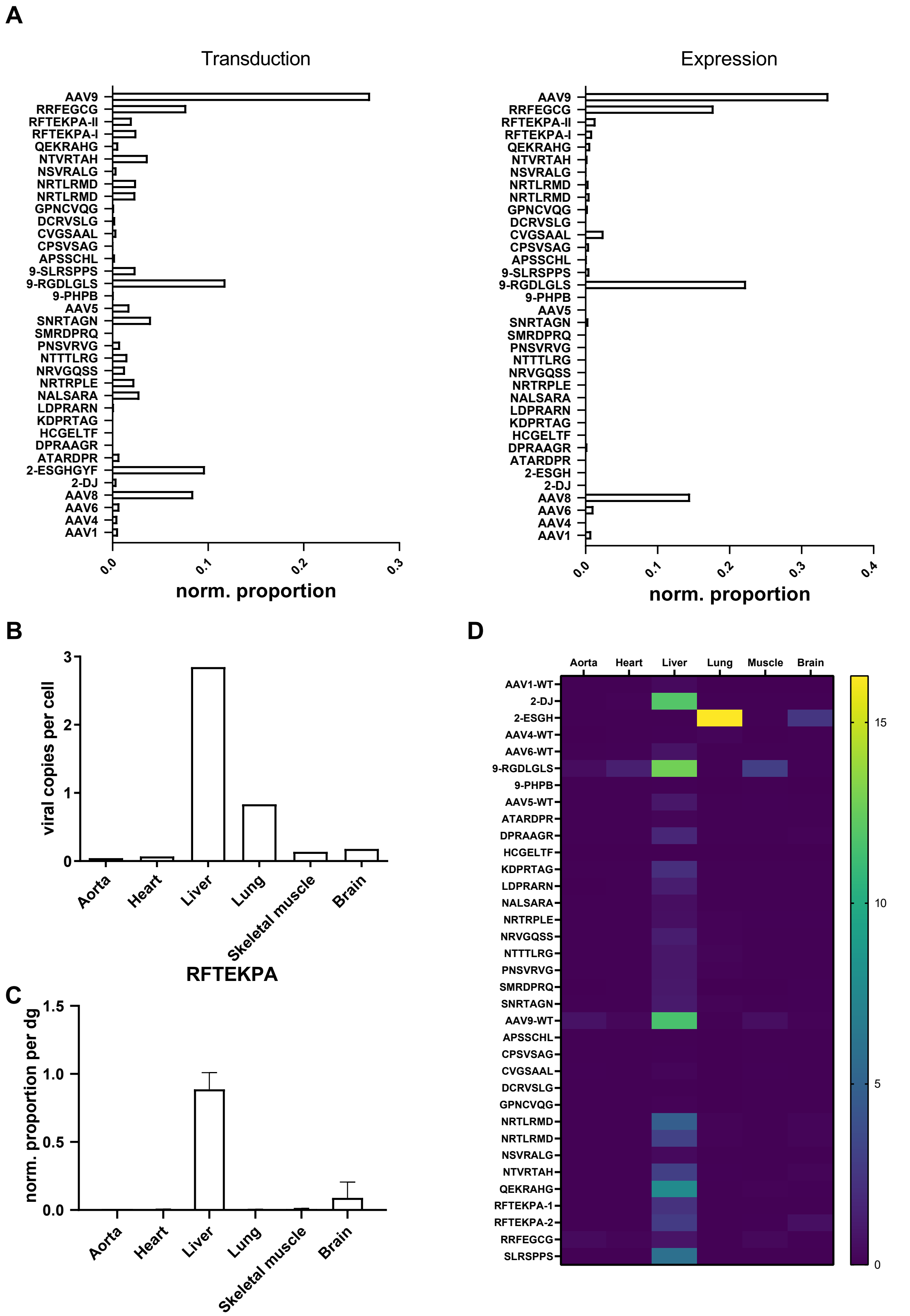

A
Transduction
AAV9
RRFEGCG
RFTEKPA-II
RFTEKPA-I
QEKRAHG
NTVRTAH
NSVRALG
NRTLRMD
NRTLRMD
GPNCVQG
DCRVSLG
CVGSAAL
CPSVSAG
APSSCHL
9-SLRSPPS
9-RGDLGLS
9-PHPB
AAV5
SNRTAGN
SMRDPRQ
PNSVRVG
NTTTLRG
NRVGQSS
NRTRPLE
NALSARA
LDPRARN
KDPRTAG
HCGELTF
DPRAAGR
ATARDPR
2-ESGHGYF
2-DJ
AAV8
AAV6
AAV4
AAV1
0.0
0.1
0.2
0.3
norm. proportion
Expression
AAV9
RRFEGCG
RFTEKPA-II
RFTEKPA-I
QEKRAHG
NTVRTAH
NSVRALG
NRTLRMD
NRTLRMD
GPNCVQG
DCRVSLG
CVGSAAL
CPSVSAG
APSSCHL
9-SLRSPPS
9-RGDLGLS
9-PHPB
AAV5
SNRTAGN
SMRDPRQ
PNSVRVG
NTTTLRG
NRVGQSS
NRTRPLE
NALSARA
LDPRARN
KDPRTAG
HCGELTF
DPRAAGR
ATARDPR
2-ESGH
2-DJ
AAV8
AAV6
AAV4
AAV1
0.0
0.1
0.2
0.3
0.4
norm. proportion
B
viral copies per cell
3
2
1
0
Aorta
Heart
Liver
Lung
Skeletal muscle
Brain
C
RFTEKPA
norm. proportion per dg
1.5
1.0
0.5
0.0
Aorta
Heart
Liver
Lung
Skeletal muscle
Brain
D
Aorta
Heart
Liver
Lung
Muscle
Brain
AAV1-WT
2-DJ
2-ESGH
AAV4-WT
AAV6-WT
9-RGDLGLS
9-PHPB
AAV5-WT
ATARDPR
DPRAAGR
HCGELTF
KDPRTAG
LDPRARN
NALSARA
NRTRPLE
NRVGQSS
NTTTLRG
PNSVRVG
SMRDPRQ
SNRTAGN
AAV9-WT
APSSCHL
CPSVSAG
CVGSAAL
DCRVSLG
GPNCVQG
NRTLRMD
NRTLRMD
NSVRALG
NTVRTAH
QEKRAHG
RFTEKPA-1
RFTEKPA-2
RRFEGCG
SLRSPPS
15
10
5
0

Figure S2. Analysis of barcoded capsid library containing several *in vitro* selected capsids motifs and benchmark AAVs in mice after systemic (i.v.) delivery. Reads of individual barcoded genomes are depicted as normalized proportion. (A) Comparison of transduction and gene expression of distinct variants in murine aorta. No novel capsid variant led to increased transduction and transgene expression in aorta compared to AAV9 wild-type. (B) Determination of viral genomes of RFTEKPA in different organs. The majority of all viral genomes ended up in the liver. (C) The specificity score based on normalized proportion of reads per diploid genome of RFTEKPA was calculated for representative organs and indicates accumulation only in the liver. (D) Heatmap of barcode expression of different AAV variants in representative organs indicating lack of efficiency of individual capsid motifs in murine aorta.

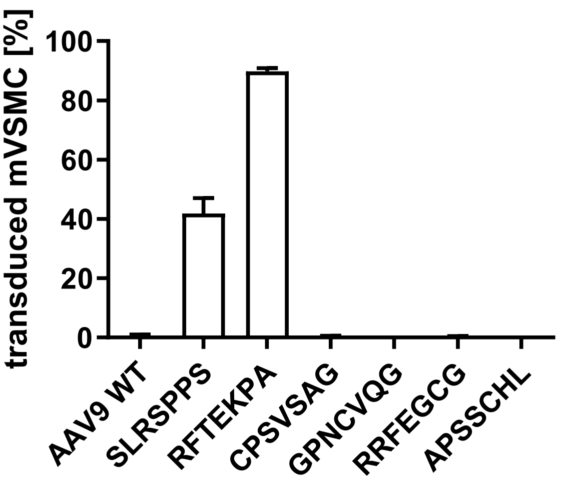

Figure S3. Characterization of the capsid motifs in mVSMCs in flow cytometry. SLRSPPS and RFTEKPA mediated transduction in mVSMCs, but not the further motifs that accumulated over the selection process (CPSVSAG, GPNCVQG, APSSCHL, technical replicates).

Table S1. Comparison of enriched motifs after 3 selection rounds on DNA and RNA level. Peptide motifs occurring in both libraries (SLRSPPS, RFTEKPA, and APSSCHL) are highlighted.

	DNA			RNA		
Rank	nt sequence (5' → 3')	%	peptide motif	nt sequence (5' → 3')	%	peptide motif
1	AGCCTGCGGAGCCCACCTAGC	6,3	**SLRSPPS**	AGCCTGCGGAGCCCACCTAGC	37,6	**SLRSPPS**
2	CGTTTTACTGAGAAGCCTGCT	5,5	**RFTEKPA**	CGTTTTACTGAGAAGCCTGCT	34,5	**RFTEKPA**
3	TGTCCTAGTGTGTCTGCGGGG	1,6	CPSVSAG	GCTCCGTCGTCGTGTCATCTT	2,3	**APSSCHL**
4	GGTCCTAATTGTGTTCAGGGG	1,4	GPNCVQG	ATGTCGAGTTGTTCGTTGGGG	2,1	MSSCSLG
5	CGGAGGTTTGAGGGGTGTGGG	1,4	RRFEGCG	GCGCCGGTGCAGCGGTATGGT	1,5	APVQRYG
6	GGTTTGCCTCAGGCGTTTTTG	1,3	GLPQAFL	ACTTGTCATACGCAGCTGCCT	1,5	TCHTQLP
7	TTGCAGCCGCAGGCGGTGCGG	1,2	LQPQAVR	CTTTCTGGGCTGCTTACGACT	1,4	LSGLLTT
8	GCTCCGTCGTCGTGTCATCTT	1,1	**APSSCHL**	GGTTCTCTGGGGTTTTTGGGT	1,3	GSLGFLG
9	TCGCATTTTCCGAGGGCGATT	1,0	SHFPRAI	AGGCCGGCGATTTCTTTGCCG	1,2	RPAISLP
10	TTGGCGGCTTCTTGGGATCCT	0,9	LAASWDP	AATGCTTGTCGGCAGCAGCTG	1,2	NACRQQL
